# Association between health-related quality of life and menopausal status and symptoms in women living with HIV aged 45–60 years in England: An analysis of the PRIME study

**DOI:** 10.1177/17455065211068722

**Published:** 2022-01-13

**Authors:** Hajra Okhai, Livia Dragomir, Erica RM Pool, Caroline A Sabin, Alec Miners, Lorraine Sherr, Katharina Haag, Rageshri Dhairyawan, Nina Vora, Binta Sultan, Richard Gilson, Fiona Burns, Yvonne Gilleece, Rachael Jones, Frank Post, Jonathan Ross, Andrew Ustianowski, Shema Tariq

**Affiliations:** 1 Institute for Global Health, University College London, London, UK; 2 National Institute for Health Research (NIHR) Health Protection Research Unit in Blood Borne and Sexually Transmitted Infections, University College London, London, UK; 3 Centre for Ageing Better, London, UK; 4 Mortimer Market Centre, Central North West London NHS Foundation Trust, London, UK; 5 Department of Health Services Research and Policy, London School of Hygiene and Tropical Medicine, London, UK; 6 Barts Health NHS Trust, London, UK; 7 Blizard Institute, Queen Mary University of London, London, UK; 8 Royal Free London NHS Foundation Trust, London, UK; 9 Lawson Unit, Brighton & Sussex University Hospitals NHS Trust, Brighton, UK; 10 Chelsea and Westminster Hospital NHS Foundation Trust, London, UK; 11 King’s College Hospital NHS Foundation Trust, London, UK; 12 University Hospital Birmingham NHS Foundation Trust, Birmingham, UK; 13 Manchester University NHS Foundation Trust, Manchester, UK

**Keywords:** HIV, menopause, quality of life, women

## Abstract

**Objectives:**

The aim of this study was to compare the health-related quality of life between mid-life women with HIV and the general population and to investigate the association between health-related quality of life and menopausal (1) status and (2) symptoms among women with HIV.

**Methods:**

Cross-sectional data of women with HIV aged 45–60 years from the Positive Transitions Through the Menopause Study. Health-related quality of life was assessed using the Euroqol questionnaire with utility scores categorizing health as perfect (score = 1.00), sub-optimal (0.75–0.99) or poor (< 0.75). Scores were compared between Positive Transitions Through the Menopause study participants and women (aged 45–59 years) from the Health Survey for England. Associations between health-related quality of life and menopausal status/symptoms in Positive Transitions Through the Menopause participants were assessed using a multivariable two-part regression model, the results of which are combined to produce a single marginal effect.

**Results:**

In total, 813 women from the Positive Transitions Through the Menopause study were included (median age 49 (interquartile range: 47–53) years); the majority were of Black African ethnicity (72.2%). Overall, 20.9%, 43.7% and 35.3% of women were pre-, peri- and post-menopausal, respectively, and 69.7% experienced mild/moderate/severe menopausal symptoms. Approximately, 40% reported perfect health, 22.1% sub-optimal health and 39.0% poor health, similar to women from the Health Survey for England (perfect health: 36.9%, sub-optimal health: 25.2%, poor health: 37.9%). In multivariable models, we found an association between health-related quality of life and peri-menopausal status (marginal effect: 0.07 (0.02, 0.12)); however, the association with post-menopausal status was attenuated (marginal effect: 0.01 (–0.05, 0.06)). There remained a strong association between lower utility scores and moderate (marginal effect: 0.16 (0.11, 0.20)) and severe (marginal effect: 0.32 (0.27, 0.39)) menopausal symptoms.

**Conclusion:**

There were no differences in health-related quality of life between women with HIV (Positive Transitions Through the Menopause participants) and women from the Health Survey for England dataset. Among Positive Transitions Through the Menopause participants, health-related quality of life was reduced in peri-menopausal women and those with increasingly severe menopausal symptoms. Our findings highlight the importance of proactive assessment of menopausal status and symptoms to optimize health-related quality of life in women living with HIV as they reach mid-life and beyond.

## Introduction

Increasing availability of effective antiretroviral therapy (ART) means that women with HIV are living longer and are experiencing age-related events, such as menopause.^1^ In the United Kingdom, the number of women living with HIV of potentially menopausal age (45–56 years) rose fivefold to 11,000 between 2008 and 2018.^2^ Women with HIV already face a myriad of intersecting challenges that impact on their quality of life (QoL), including gender-based violence, poverty, poor mental health and relationship difficulties.^3^ There is limited information available on the additional impact of menopause on the QoL of these women.

Menopause is driven by a decline in ovarian function, leading to oestrogen depletion, and is defined as the absence of menses for at least 12 months (with women categorized as post-menopausal beyond this time).^4^ This is preceded by peri-menopause, characterized by significant hormonal fluctuations, menstrual irregularity and menopausal symptoms, which can last for several years.^4^ The menopause transition impacts a wide range of physical and psychological functions and is often accompanied by significant social transitions as a result of ageing, such as caring responsibilities for older parents and children leaving home.^5^

Health-related Quality of life (HR-QoL) is the subjective evaluation of one’s personal well-being in multiple domains, including physical, social and psychological.^6^ Data from Public Health England have shown that women living with HIV have poorer HR-QoL than women in the general English population, scoring lower in every domain, with anxiety and depression being particularly prevalent.^7^ Therefore, the menopause transition may present an additional challenge to this population, particularly as women living with HIV been documented to experience high prevalence of menopausal symptoms.^8–11^

Menopause is recognized to have negative and multidimensional impacts on HR-QoL, with vasomotor and urogenital symptoms particularly associated with diminished HR-QoL.^12–17^ However, the association between menopause and HR-QoL is complex and dynamic; peri-menopausal women (who are likely to be experiencing a peak in their symptoms) are more likely than post-menopausal women to experience reduced HR-QoL, particularly if their symptoms are prolonged.^15^ However, despite clear evidence that menopause impacts HR-QoL in the general population, and that women with HIV already experience reduced HR-QoL, we lack data evaluating the combined effects of menopausal status and symptoms, and living with HIV on HR-QoL.

Drawing on data from the Positive Transitions Through the Menopause Study (PRIME), we compare HR-QoL in a sample of women living with HIV aged 45–60 years in England with similarly aged women from the general population in the Health Survey for England (HSE) dataset. Among PRIME participants (all women with HIV) only, we further investigate the association between HR-QoL and (1) menopausal status and (2) menopausal symptoms.

## Methods

The PRIME Study was a cross-sectional, mixed-methods observational study of the impact of the menopause transition on the health and well-being of women with HIV. The study methods are described in detail elsewhere.^18^ Briefly, women aged between 45 and 60 years were recruited from one of the 21 National Health Service (NHS) HIV clinics across England between February 2016 and June 2017. Women were ineligible if they had experienced surgical menopause, had received chemotherapy or radiotherapy in the last 6 months, or if their last menstrual period was more than 60 months prior to study enrolment. The PRIME Study had ethical approval from the South East Coast-Surrey Research Ethics Committee (REF 15/0735). All participants provided written informed consent.

Participants were asked to self-complete paper questionnaires comprising questions relating to demographic/social factors (age, ethnicity, employment status, relationship status, highest level of education), current lifestyle factors (smoking, recreation drug use, alcohol use (assessed using the Alcohol Use Disorders Identification Test, AUDIT-C, with a score of > 5 considered to be risky)), HIV-related and non-HIV medical history (year of HIV diagnosis, history of depression, diabetes, hypertension, cardiovascular disease, hepatitis B/C co-infection, breast cancer, osteoporosis, stroke) and menopause-related symptoms (measured using the validated menopause rating scale (MRS)). The total number of medical conditions reported by women (from the list above) was used as a proxy for multimorbidity. CD4 + T-cell count and HIV viral load (VL) were obtained from clinical records (where women consented to this), with missing values completed through self-report. Last HIV VL was categorized as ‘undetectable’ if < 50 copies/mL in clinical data or, if missing, reported as ‘undetectable’ by participants in the questionnaire.

Menopausal status was determined from self-reported menstrual pattern (without biological confirmation) and was categorized as follows: pre-menopausal (reporting regular menstruation), peri-menopausal (reporting irregular periods over the previous 2 years) and post-menopausal (⩾ 12 months amenorrhoea).

The MRS measures the perceived severity of 11 symptoms from three domains: somatic (episodes of hot flashes/sweating, heart discomfort, sleeping disorders and joint/muscle complaints), psychological (depression, irritability, anxiety and exhaustion) and urogenital (sexual problems, vaginal dryness and urinary complaints). For each symptom, perceived severity was scored on a Likert-type scale from 0 (*symptoms not present*) to 4 (*very severe*). A composite score was calculated by summing the scores and was categorized as: *no/few* (0–4), *mild*,^5–8^ *moderate*^9–16^ and *severe* (⩾ 17) symptoms.^19^

HSE data^20^ were used descriptively to compare the HR-QoL of women from the PRIME study to the general population. HSE is designed to measure health and health-related behaviours in people living in a random sample of private households in England; it excludes people who are homeless or living in communal buildings, such as nursing homes. Participants are interviewed at home and later visited by a nurse to collect more detailed clinical information. The 2018 survey included interviews for 8178 people aged 16 years or above. Information about HIV status is not recorded. For the purpose of this analysis, we selected only women aged between 45 and 59 years (n = 1067) as a direct comparison to women recruited in the PRIME dataset. Data were accessed from the UK Data Service.^21^

HR-QoL was ascertained through the Euroqol questionnaire (EQ-5D-3L) instrument.^22^ The EQ-5D-3L is a generic classification system that measures current health in five domains (mobility, self-care, ability to do usual activities, pain or discomfort and anxiety/depression), each with three levels (indicating no, some or extreme problems), with the availability of country-specific utility algorithms.^23^ Utilities are measured on interval scales, for which perfect health (no problems on all five domains) is equivalent to a value of 1 and death is equivalent to a value of 0 (negative values can be scored and are valued as worse than death).

Demographic/social, lifestyle and clinical characteristics of women included were described using number/percentage or median/interquartile range (IQR) for categorical and continuous variables, respectively.

Each of the five domains explored in the EQ-5D-3L instrument was presented as numbers/percentages. EQ-5D-3L utility score data were non-normally distributed, with a spike at 1.0 (suggesting a high proportion of individuals reporting perfect health). Therefore, for descriptive purposes, we describe the EQ-5D-3L utility score in three groups: 1.00, perfect health; 0.75–0.99, sub-optimal health; and < 0.75, poor health.

To address the skewed nature of the data, a two-part regression model (as reported previously by Miners et al.^24^) was used to explore the association with menopausal status and symptoms (MRS total score, somatic symptoms score, urogenital symptoms score and psychological symptoms score) among women from the PRIME study. This involved fitting two regression models, the results of which are combined to produce a single measure of effect (known as a marginal effect (ME)) on EQ-5D-3L utility score. First, a logistic regression was done, in which the dependent variable indicated perfect health (yes or no). Generalized linear modelling was then done for the data relating to people with less than perfect health, with a γ distribution and log link because of the left skewed nature of the data. Data were modelled using the -twopm- command developed for STATA v16.^25^ As this methodology was developed for healthcare cost data, the utility score produced from the EQ-5D-3L utility score was reversed (perfect health = 0 and death = 1); therefore, in this analysis a negative ME estimate indicates better health, whereas a positive value indicates poorer health.

These models were adjusted for characteristics known to be associated with both HR-QoL and menopausal status and/or symptoms (from published literature) which were selected a priori. These included age, ethnicity, employment status, marital status, education status, having enough money for basic needs, smoking status, alcohol consumption, number of medical conditions, years since HIV diagnosis, last available CD4+ T-cell count and HIV VL.

## Results

### Study participants

*PRIME Study*: Of the 868 women included in the PRIME study, 813 (93.7%) completed all questions related to HR-QoL (Table 1). When compared to the women included in this analysis, those who did not complete the questionnaire were less likely to have a university education (12/55; 29.3%) and more likely to report not having enough money for basic needs (12/55; 24.0%). A higher proportion of women with missing data on HR-QoL had a detectable HIV VL (9/55; 19.1%) at their last assessment and nadir CD4+ T-cell count of < 200 cells/mm^3^ (23/30; 76.7%) where available.

**Table 1.** Demographic, social, lifestyle and clinical characteristics of PRIME participants and mid-life women from the HSE data (general population sample) who completed the EQ-5D-3L instrument.

		PRIME women N (%)	HSE^ women N (%)
		813	1067
Age (years)	45–49	382 (50.2)	347 (32.5)
	50–54	291 (38.2)	369 (34.6)
	55–59	88 (11.6)	351 (32.9)
Ethnicity	Black any	642 (81.2)	43 (4.0)
	White UK	99 (12.5)	941 (88.3)
	Other	50 (6.3)	82 (7.7)
Employment status	Full-/Part-time Employment	525 (67.1)	847 (79.5)
	No employment	257 (32.9)	219 (20.5)
Relationship status	No relationship	357 (46.9)	299 (28.0)
	Married	158 (20.7)	644 (60.4)
	Cohabiting	247 (32.4)	124 (11.6)
Education status	No qualifications	86 (11.2)	115 (10.8)
	Lower than university level	336 (43.7)	628 (58.9)
	University	347 (45.1)	321 (30.2)
Enough money for basic needs	All of the time	300 (37.3)	–
	Most of the time	216 (26.8)	–
	Some of the time	209 (26.0)	–
	Never	80 (9.9)	–
Household income tertiles (£)	⩽19,500	–	353 (39.3)
	19,501–38,356	–	286 (31.9)
	>38,356	–	259 (28.8)
Current smoker		68 (8.6)	189 (17.7)
Recreational drug use in last 3 months		20 (2.5)	–
Risky alcohol use (AUDIT-C score > 5)		124 (15.3)	154 (14.4)
Number of medical conditions*	0	521 (64.1)	–
	1	219 (26.9)	–
	2	62 (7.6)	–
	>3	11 (1.4)	–
Menopausal symptoms	None/little	204 (30.3)	–
	Mild	110 (16.3)	–
	Moderate	174 (25.8)	–
	Severe	186 (27.6)	–
Years since HIV diagnosis	<10	192 (25.3)	–
	>10	566 (74.7)	–
Last HIV viral load	Undetectable	679 (88.5)	–
	Detectable	88 (11.5)	–
Last CD4 + T-cell count (cells/mm^3^)	>500	490 (68.2)	–
	200–500	182 (25.3)	–
	<200	47 (6.5)	–

%: percentage; IQR: interquartile range. ^ Health Survey England, 2018 data (restricted to women aged 45–59 years). * in addition to HIV.

Missing: Ethnicity: 22; Employment status: 31; Relationship status: 51; Education status: 44; Enough money for basic needs: 8; Current smoker: 19; Recreational drug use: 20; Menopausal symptoms: 139; Last HIV viral load: 46; Last CD4 + T-cell count: 94.

Among the women included (Table 1), the median age was 49 (IQR: 47–53) years. The majority were of Black African ethnicity (571; 72.2%) and were born outside the United Kingdom (681; 85.0%). A large proportion of women had completed university education (347; 45.1%); approximately two-thirds reported being employed (full-time: 391; 50.0%/part-time: 134; 17.1%) and (516; 64.1%) reported having enough money to meet their basic needs all or most of the time. Only 68 (8.6%) and 20 (2.5%) women reported smoking and recreational drug use, respectively. Approximately, one in three women had at least one medical condition in addition to HIV (292; 35.9%).

Three-quarters of women had been living with HIV for 10 years or more; almost all were currently on ART (768; 97.6%), two-thirds had a CD4 + T-cell count > 500 cells/mm^3^ (490; 68.2%) and most had an undetectable HIV VL at their last available measure (697; 88.5%).

Overall, 20.9%, 43.7% and 35.3% of women were classified as pre-, peri- and post-menopausal, respectively. Overall, approximately two-thirds experienced mild/moderate/severe menopausal symptoms (mild: 16.3%; moderate: 25.8%; severe: 27.6%) based on the MRS scale. This was similar across all three symptoms domains (*somatic* – mild: 18.8%; moderate: 30.1%; severe: 16.2%; *psychological* – mild: 16.8%; moderate: 18.0%; severe: 26.8%; *urogenital* – mild: 11.6%; moderate: 23.7%; severe: 29.2%).

*HSE dataset*: Of the 5562 women included in the HSE dataset, 1170 women were aged between 45 and 59 years. Of these, 91.2% (1067) completed the HR-OoL questions. In contrast to women from the PRIME study, HSE women were more likely to be of a White ethnic origin (HSE: 88.3% versus PRIME: 12.5%), be in some form of employment (HSE: 79.5% versus PRIME 67.1%) and be married (HSE: 60.4% versus PRIME: 20.7%). However, HSE participants were less likely to have attained a university level education in comparison to women from the PRIME dataset (HSE: 30.2% versus PRIME: 45.1%). Over a quarter (28.8%) women from the HSE dataset report living in the lowest household income tertiles compared to 35.9% of PRIME women who report not having enough money for basic needs (however important to note these measures are not directly comparable).

### HR-QoL

Of the five EQ-5D-3L domains, approximately one-quarter of women from PRIME reported at least some problems with mobility (209; 25.7%) and performing their usual activities (191; 23.5%), with the least impact being reported on self-care (90; 11.1%). Although this was similar among women from HSE (mobility: 265, 24.8%; usual activities: 257, 24.0%; self-care: 10.6%), a much higher proportion of women from the HSE reported extreme problems in all three of these domains when compared to PRIME women (Table 2). A large proportion of women living with HIV reported experiencing at least some pain (426; 52.4%) and anxiety/depression (334; 39.9%). This was similar among women from the general population in the HSE dataset (pain: 579, 54.3%; anxiety: 399, 37.4%).

**Table 2.** QoL domains reported in the EQ-5D-3L instrument in women living with HIV (PRIME) and mid-life women from HSE (general population sample).

		PRIME N (%)	HSE^ N (%)	p-value
		813	1067	
Mobility	No problems	604 (74.3)	802 (75.2)	<0.001
	Some problems	207 (25.5)	208 (19.5)	
	Extreme problems	2 (0.2)	57 (5.3)	
Self-care	No problems	723 (88.9)	954 (89.4)	0.002
	Some problems	86 (10.6)	89 (8.3)	
	Extreme problems	4 (0.5)	24 (2.2)	
Usual activity	No problems	622 (76.5)	810 (75.9)	0.003
	Some problems	172 (21.2)	200 (18.7)	
	Extreme problems	19 (2.3)	57 (5.3)	
Pain/discomfort	No problems	387 (47.6)	488 (45.7)	0.71
	Some problems	359 (44.2)	491 (46.0)	
	Extreme problems	67 (8.2)	88 (8.2)	
Anxiety/depression	No problems	489 (60.1)	668 (62.6)	0.36
	Some problems	274 (33.7)	347 (32.5)	
	Extreme problems	50 (6.2)	52 (4.9)	
EQ-5D-3L utility score	Mean (SD)	0.75 (0.30)	0.74 (0.35)	0.44
	Median (IQR)	0.80 (0.69, 1.00)	0.80 (0.69, 1.00)	0.98

^HSE, 2018 data (restricted to women aged 45–59 years).

The overall median EQ-5D-3L utility score among mid-life women living with HIV suggested sub-optimal health (median: 0.8; range: −0.3, 1.0). This was similar to the median EQ-5D-3L utility score in mid-life women from the HSE in 2018 (p = 0.98; Table 2).

After categorizing the utility score in PRIME participants, 38.9% (316) had an EQ-5D-3L utility score of 1.00 (i.e. perfect health); 22.1% (180) had an EQ-5D-3L utility score between 0.75 and 0.99 (i.e. sub-optimal health); and 39.0% (317) had an EQ-5D-3L utility score < 0.75 (i.e. poor health), of whom 15% (45) had an EQ-5D-3L utility score of less than 0 (i.e. worse than death). This was broadly similar to the distribution of EQ-5D-3L scores among women from HSE (perfect health: 36.9%; sub-optimal health: 25.2%; poor health: 37.9%; p = 0.30).

### Menopausal status/symptoms and HR-QoL in PRIME participants

**Figure 1.**
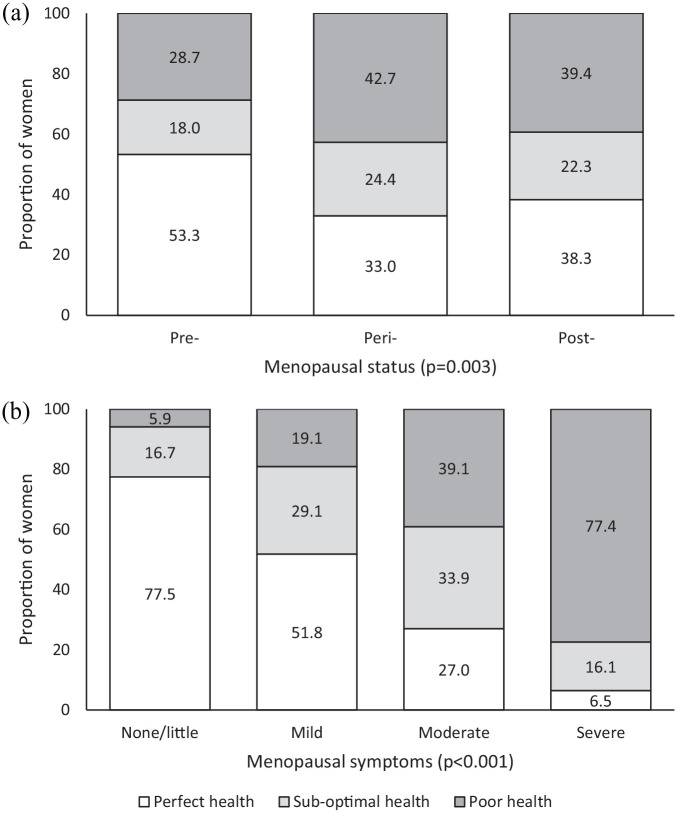
Categorical EQ-5D-3L score by (a) menopausal status (n = 798) and (b) severity of menopausal symptoms (n = 674) among PRIME participants.

Menopausal status was available for 98.2% (n = 798) of women included in this analysis. There were no systematic differences between the women for whom such data were and were not available. A lower proportion of peri- and post-menopausal women living with HIV reported perfect health when compared to pre-menopausal women (pre-menopausal: 52.3%, peri-menopausal: 33.0%, post-menopausal: 38.3%). The proportions of peri- and post-menopausal women reporting sub-optimal/poor health were similar (Figure 1(a)).

Among women with available menopausal symptom data (82.9%, n = 674), the proportion reporting sub-optimal/poor health dramatically increased as the severity of symptoms increased (Figure 1(b)). Women who did not complete the MRS (and who are therefore excluded from this analysis) were more likely to be unemployed (excluded: 45.0% versus included: 30.4%), were not currently in a relationship (69.2% versus 42.8%) and did not have enough money for basic needs (16.3% versus 8.7%).

Similar to the crude analysis, the ME of menopausal status on EQ-5D-3L utility score of peri-menopausal status on EQ-5D-3L utility score was 0.08 (95% confidence interval: 0.03, 0.14) compared to pre-menopausal women. That is, peri-menopausal women were likely to have a mean EQ-5D-3L utility score 0.08 *lower* than those who were pre-menopausal. This was similar for post-menopausal women (ME post-menopausal: 0.08 (0.02, 0.13) versus pre-menopausal). The ME estimates were larger and more significantly associated with severity of menopausal symptoms (ME mild: 0.07 (0.02, 0.12); ME moderate: 0.17 (0.13, 0.21); ME severe: 0.42 (0.38, 0.47) versus none/little). In other words, women with mild, moderate and severe menopausal symptoms were likely to have a mean EQ-5D-3L utility score 0.07, 0.16 and 0.42 *lower* than those without symptoms, respectively. This pattern was similar for all three individual MRS domains (Table 3).

**Table 3.** Marginal effects of menopausal status and symptoms on EQ-5D-3L score.

	Unadjusted ME (95% CI)	p-value	Adjusted^a^ ME (95% CI)	p-value
Menopausal status				
Pre-menopause	Ref.	0.0001	Ref.	0.0002
Peri-menopause	0.08^ (0.03, 0.14)		0.07 (0.02, 0.12)	
Post-menopause	0.08 (0.02, 0.13)		0.01 (−0.05, 0.06)	
Menopausal symptoms				
None/little	Ref.	<0.0001	Ref.	<0.0001
Mild	0.07 (0.02, 0.12)		0.06 (0.01, 0.11)	
Moderate	0.17 (0.13, 0.21)		0.16 (0.11, 0.20)	
Severe	0.42 (0.38, 0.47)		0.32 (0.27, 0.39)	
Somatic symptoms				
None/little	Ref.	<0.0001	Ref.	<0.0001
Mild	0.09 (0.05, 0.14)		0.10 (0.05, 0.15)	
Moderate	0.24 (0.20, 0.29)		0.20 (0.15, 0.24)	
Severe	0.42 (0.36, 0.48)		0.31 (0.24, 0.37)	
Urogenital symptoms				
None/little	Ref.	<0.0001	Ref.	<0.0001
Mild	0.06 (−0.01, 0.12)		0.08 (0.02, 0.15)	
Moderate	0.15 (0.10, 0.20)		0.10 (0.05, 0.15)	
Severe	0.23 (0.17, 0.28)		0.19 (0.14, 0.24)	
Psychological symptoms				
None/little	Ref.	<0.0001	Ref.	<0.0001
Mild	0.11 (0.06, 0.16)		0.11 (0.06, 0.16)	
Moderate	0.16 (0.11, 0.21)		0.14 (0.09, 0.19)	
Severe	0.42 (0.37, 0.46)		0.32 (0.27, 0.38)	

a Adjusted for age, ethnicity, employment status, marital status, education status, enough money for basic needs, smoking status, alcohol consumption, number of medical conditions, years since HIV diagnosis, last available CD4 + T-cell count and VL measure.

^Interpretation: peri-menopausal women are likely to have a mean EQ-5D-3L utility score 0.08 *lower* than women in the pre-menopausal stage.

After adjustment for the a priori confounders described earlier, there remained evidence of lower EQ-5D-3L utility scores among peri-menopausal women (ME peri-menopausal: 0.07 (0.02, 0.12)). However, the association with post-menopausal status was attenuated (ME post-menopausal: 0.01 (−0.05, 0.06)). In this model, lower EQ-5D-3L utility scores were also associated with unemployment (versus full-time employment), not having money for basic needs (versus always having money for basic needs), current smoking (versus not smoking), having additional medical conditions to HIV (versus no additional medical conditions), and having been diagnosed with HIV ⩾ 10 years ago (versus being diagnosed < 10 years ago).

In contrast, there remained a strong association between lower EQ-5D-3L utility scores and moderate (ME moderate: 0.16 (0.11, 0.20)) and severe (ME severe: 0.32 (0.27, 0.39)) menopausal symptoms in adjusted models. Similar to the menopausal status model, there were also associations with unemployment, not having money for basic needs, current smoking, being diagnosed with HIV for ⩾ 10 years, and last CD4 + T-cell count of 200–500 cells/mm^3^ (versus > 500 cells/mm^3^). This association also remained for all MRS symptom domains; however, somatic and psychological symptoms were associated with a greater reduction of HR-QoL in comparison to urogenital symptoms (Table 3).

## Discussion

In this analysis of data from women aged 45–60 years attending HIV clinics in England, we observed reductions in HR-QoL in peri- and post-menopausal women, and in those with increasingly severe menopausal symptoms. This analysis builds on previous work on HR-QoL in women living with HIV by specifically examining the association with menopause and demonstrates the importance of ascertaining menopausal status and identifying menopausal symptoms in women living with HIV. We report that women who were peri-menopausal (a time when menopausal symptoms peak) had substantially lower HR-QoL scores compared to pre-menopausal women. This association was also observed for post-menopausal women but was attenuated in the adjusted analysis. Increasing severity of menopausal symptoms was strongly associated with poorer HR-QoL; women living with HIV with severe menopausal symptoms had an estimated mean EQ-5D-3L utility score of 0.33 lower than that among women with few or no menopausal symptoms. To put this into context, in their analysis of HR-QoL among people living with HIV, Miners et al.^24^ found that people living with HIV had a utility score of 0.11 lower than those without HIV, one-third of the reduction that we have observed with menopausal symptoms.

In contrast to the well-documented associations between HIV-positive status and reduced HR-QoL,^24,26^ we found that mean HR-QoL scores for women living with HIV were similar to those of women of the same age in the general population (using HSE data). This suggests that the average HR-QoL score is less than optimal for both groups of women, regardless of HIV status, and may be a result of ageing. It is important to note that the majority of PRIME Study participants had an undetectable VL and a CD4 + T-cell count > 500 cells/mm^3^, indicating that this is a population that is adherent to ART with well-controlled HIV. Furthermore, the similarities in HR-QoL scores between women with and without HIV in this analysis may suggest that these women living with HIV remain resilient despite the additional challenge of living with HIV. Nonetheless, there are substantial differences between the two populations which must be considered when they are compared. For example, 81% of PRIME participants were of Black African or other Black ethnicities, whereas the majority of women in HSE were White (88.2%; 20) and a higher proportion of women from the PRIME study had a university education (PRIME: 45.1%; HSE: 30.2%) or were not in full- or part-time employment (PRIME: 32.9%; HSE: 20.5%).

It is estimated that 85% of women experience at least one symptom during the peri-menopause, with symptoms predating the final menstrual period and potentially persisting for several years post-menopause.^27^ The most prevalent symptoms are vasomotor (such as hot flushes and night sweats); however, urogenital symptoms (including vaginal dryness, urinary symptoms and sexual dysfunction), mood changes, disrupted sleep, cognitive changes and pain are also commonly reported.^28^ Among women with HIV, it has been reported that the prevalence of menopausal symptoms is high and includes hot flushes, pain, mood changes, fatigue and sleep disturbance.^8,9,29–31^ With further evidence to suggest that the severity of vasomotor and psychological symptoms of menopause is greater in women living with HIV than those without.^32,33^ Although we know that hormone replacement therapy (HRT) and topical vaginal oestrogens alleviate menopausal symptoms and improve women’s HR-QoL,^34,35^ use of both treatment modalities remains low among women living with HIV, as has been previously reported in the PRIME study.^8^ Furthermore, women with HIV report difficulties accessing appropriate menopause care,^31^ while primary care providers have been shown to have low levels of confidence in managing menopause in this patient group.^36^

Supporting women living with HIV to have the optimal QoL as they age requires holistic management of menopausal symptoms. The impact of menopausal status and symptoms on HR-QoL demonstrated in this study highlights the importance of clear clinic pathways and national guidance on menopause for women living with HIV to ensure high quality care for this growing patient group.^37^ Community generated resources are also key to improving awareness among women; one such example is the ‘We Are Still Here’ campaign by the UK-based HIV advocacy charity Sophia Forum^38^ which includes an accessible booklet on HIV and menopause specifically targeted to this patient group.

EQ-5D-3L is a widely used instrument, which measures HR-QoL in multiple domains, with robust evidence of construct and convergent validity.^39,40^ It has been used frequently in HIV research and has been shown to have good psychometric properties when measuring HR-QoL among people with HIV.^41,42^ Using EQ-5D-3L has allowed us to compare HR-QoL with a general population sample (HSE) deploying the same measure and affords us the opportunity to undertake comparative analysis with other HIV datasets in the future. However, it is important to remember that EQ-5D-3L is a self-reported measure based on how a person is feeling on that particular day. It is therefore not sensitive to fluctuations in symptoms, a common feature of menopause. In addition, it may not capture specific HIV-related factors which impact QoL in people living with HIV, such as stigma and discrimination, and side effects of ART.

This analysis is the largest to date in the United Kingdom to explore HR-QoL among mid-life women living with HIV, an increasingly important patient group. It is also the first study internationally to specifically examine the association between HR-QoL and menopausal status and menopausal symptoms. The sample is broadly representative of women living with HIV within the United Kingdom and generates important insights into HR-QoL in women living with HIV of menopausal age. However, our findings may not be generalizable to other settings where the prevalence of virological failure and behavioural risk factors, such as drug use, is higher.

As the PRIME Study is cross-sectional, we cannot determine the direction of associations and are unable to comment on changes in QoL in individuals over time. A limitation of this study is the lack of complete data on our exposures of interest (menopausal status/symptoms). However, we found few differences between women for whom data were incomplete/missing compared to those with complete data who were included in analyses. It is also important to acknowledge that we report estimates from a missing indicator analysis due to the degree of missing data. A sensitivity analysis using a complete case analysis (not shown) did not differ from the presented analyses. Menopausal status was categorized by self-reported menstrual pattern, without biological confirmation, which means that some women may have been misclassified, although this approach has been validated.^19,43^ In addition, menopausal status could act as a proxy for age, although the age range of participants in PRIME is narrow, limiting this effect. Menopausal symptoms are largely non-specific, and it can be challenging to disentangle those which are truly menopausal and those which may represent other conditions, both HIV-related and non-HIV related. Although, the MRS is internationally recognized and has been validated both in the general population and among those with multimorbidity,^19^ additional validation of the tool specifically in women living with HIV would demonstrate its ability to distinguish menopausal symptoms from those related to HIV infection. Finally, we cannot comment on whether associations between menopausal status and symptoms and HR-QoL are modified by HIV status as the HSE dataset has no information on menopausal status and symptoms.

## Conclusion

In conclusion, in this large sample of mid-life women living with HIV, we found HR-QoL was similar to that reported by women from the general population, and that peri-menopausal status and increasing severity of symptoms were both associated with poorer HR-QoL. This analysis highlights the role of reproductive ageing in the HR-QoL of women living with HIV. Our findings underline the importance of proactive assessment of menopausal status and symptoms in this patient population to provide appropriate support and management (including offering HRT and vaginal oestrogen when indicated). This is critical if we are to help women living with HIV to optimize their health as they reach mid-life and beyond.
